# Chiropractic patient compensation claims in Norway from 2013 to 2024

**DOI:** 10.1186/s12998-026-00684-6

**Published:** 2026-10-08

**Authors:** Christoffer Børsheim, Cathrine H. Feier, Carlos Gevers-Montoro, Mark Thomas, Anika Young, Jan Hartvigsen

**Affiliations:** 1 Department of Global Public Health and Primary Care, University of Bergen, Bergen, Norway; 2 Department of Kinesiology and Health Sciences, University of Waterloo, Waterloo, ON Canada; 3 Department of Psychology, McGill University, Montreal, QC Canada; 4 Alan Edwards Centre for Research on Pain, Montreal, QC Canada; 5 Department of Chiropractic, Université du Québec à Trois-Rivières, Trois-Rivières, QC Canada; 6 London South Bank University, London, UK; 7 Spinal Pain Research Centre, Department of Chiropractic, Faculty of Medicine, Health and Human Sciences, Macquarie University, Sydney, Australia; 8 Center for Muscle and Joint Health, Department of Sports Science and Clinical Biomechanics, University of Southern Denmark, Odense, Denmark; 9 Danish Institute for Advanced Study, University of Southern Denmark, Odense, Denmark; 10 Chiropractic Knowledge Hub, Odense, Denmark

**Keywords:** Patient safety, Adverse events, Medicolegal claims, Registry study, Musculoskeletal disorders

## Abstract

**Background:**

Compensation claims represent a selective subset of adverse events in healthcare and provide insight into patient safety. Compensation claims vary between countries and jurisdictions due to administrative, legal, and clinical differences. This study aimed to describe compensation claims related to chiropractic consultations in Norway from 2013 to 2024.

**Methods:**

We conducted a retrospective registry study using data from the Norwegian System of Patient Injury Compensation and national chiropractic consultation data. All completed claims associated with chiropractic care between 2013 and 2024 were included regardless of whether they were approved or rejected. Claim frequencies were calculated per 100,000 consultations and per first-assessment consultations. Descriptive analyses were used to summarise results.

**Results:**

A total of 147 claims were filed during the study period, of which 17 (12%) were approved. Over 24 million chiropractic consultations were recorded for the period, corresponding to an average annual claim approval rate of 0.1 per 100,000 consultations and 0.5 per 100,000 first-assessment consultations. Most claims involved musculoskeletal conditions (89%). High approval proportions were observed for claims related to central nervous system and circulatory disturbances (60%). The most common reasons for rejection were lack of causation and absence of healthcare failure. Total compensation amounted to 21.7 million Norwegian Kroner (~ 2.2 million Euros).

**Conclusions:**

Compensation claims related to chiropractic care in Norway are rare relative to consultation volume, and only a small proportion were approved. Most claimants presented with common musculoskeletal conditions, while approved cases often involve serious events temporally associated with chiropractic consultations. These findings contribute updated national evidence on compensation claims in chiropractic practice and may inform quality improvement and policy.

## Background

Patient safety is a priority in healthcare at policy-, system- and local levels [1–4]. Adverse events, understood as any unintended, undesirable, or unexpected response associated with a healthcare encounter [5], may occasionally result in the patient seeking compensation for alleged injury. Compensation claims therefore represent a selected subset of adverse events, as only a minority of patients who experience an adverse event initiate a claim [6]. For chiropractic care, most of the literature related to safety is focused on spinal manipulation. Adverse events following spinal manipulation are frequently (≥ 50%) reported, but the vast majority are mild, transient, and may be regarded as normal reactions (e.g. increased discomfort or soreness) [4, 7–11]. Although serious adverse events have also been reported; the existing literature primarily demonstrates temporal associations rather than established causal pathways [4, 12–14].

Chiropractors in Norway are licensed primary-contact practitioners who provide approximately two million consultations annually to 8% of the Norwegian population [15, 16]. Understanding the profile and frequency of compensation claims associated with chiropractic care in Norway is important for patient safety, professional quality improvement, and health‑system integration. Previous research on patient compensation claims from Denmark and Norway has shown that claims related to chiropractic treatment are rare, and that only a small proportion of claims result in financial compensation [17, 18]. However, no updated analysis exists that exclusively describes Norwegian chiropractic‑related claims. These previous claims studies highlighted worsening of existing pain, alleged disc herniations, and delayed referrals as the top three reasons for seeking compensation after chiropractic consultations.

The current study aims to describe all compensation claims related to chiropractic consultations in Norway from 2013 to 2024 through claim frequency, injury types, and approval rates. By providing updated national data on chiropractic-related compensation claims, this study will inform patient safety governance, professional quality improvement, and interpretation of rare but serious claims within musculoskeletal healthcare.

## Methods

### Study design

We conducted a retrospective registry study using administrative data from Norwegian System of Patient Injury Compensation (NPE, *initials in Norwegian*) and the national administrative registry for publicly funded healthcare in Norway (KUHR, *initials in Norwegian*). The study included all completed NPE cases (approved or rejected) following consultations with a chiropractor between 1 January 2013 and 31 December 2024, and annual consultation numbers for the same period.

### Setting and data

In Norway, compensation for patient injuries is administered by the NPE [19]. All licensed health personnel pay an annual premium that reflects the estimated annual cost for their profession, for 2025 the amount due per chiropractor was 6900 NOK (700 EUR). NPE manages all initial compensation claims relating to patient injury within the Norwegian healthcare system and provides a free of charge legal service. A claim is accepted only if all three of the following legal conditions are fulfilled: (1) Healthcare failure: examination, treatment, diagnosis, follow‑up, or referral below acceptable professional standard; (2) Causation: the injury is more likely than not caused by the failure; (3) Financial loss: 10’000 Norwegian kroner (NOK) (1000 EUR ± 20%) beyond any loss that would have occurred regardless of the alleged failure [20]. Throughout the manuscript, the term “injury” reflects the terminology used within the NPE classification system and does not necessarily imply a structurally defined or objectively verified injury. NPE’s case management follows a standard process involving collection of provider statements, medical records, optional patient comments, medical expert evaluation, a causation assessment, and a final decision. Processing times average 7 months for rejected claims and 11 months for accepted claims [21].

NPE claims data for Norwegian chiropractic patients in the period 2013–2024 were investigated. The dataset included patient demographics (age, sex), short case descriptions, decision year, approval status, injury category (NPE internal codes), International Classification of Disease – tenth revision (ICD‑10) codes presenting diagnosis and injury code (logged by NPE or expert consultant). Privacy legislation limited us from providing case details beyond aggregated levels. Mean and median compensation amount for the total period are provided as providing individual compensation amounts is problematic in small cohorts.

The chiropractic activity data (annual consultation volume) includes all chiropractic consultations for the period where a reimbursement claim was submitted. A small number of consultations are probably missed if claims are not submitted within the permitted billing period or if payment was outside of the reimbursement system. This data was obtained as free to access aggregated data per annum from national reimbursement data using K1 and K2 billing codes [16]. K1 represents first‑assessment consultations, which include both new patient consultations and new clinical problems for existing patients. K2 represents follow‑up consultations for existing patients. These codes allow estimation of risk at two exposure levels: claims per total consultations (K1 + K2) and claims per first‑assessment consultation (K1).

### Analysis

The analysis is descriptive, reflecting the structure of similar prior studies [17, 18], displaying annual claim counts and approval percentages. Compensation claim outcomes were described by year and injury category (defined by NPE internal codes and truncated ICD-10 codes), and adjoined free-text case comments were explored for approved cases. Furthermore, claim rates by annual Norwegian consultation volume were calculated as claims per 100,000 total consultations and per 100,000 first-assessment consultations (K1 consultations).

To preserve interpretability and enable future comparison, we chose to present our compensation claims data using NPE internal codes and ICD-10 classifications, as these reflect the original coding structure.

## Results

### Compensation claim frequency and approval rates

Between 2013 and 2024, 147 chiropractic‑related compensation claims were completed by NPE, with 17 approved, leading to an overall approval rate of 12% (Table 1). Annual counts ranged from 4 (2017) to 16 (2020 and 2022). Year‑specific approvals peaked in 2019 (4/10, 40%), while 2023 and 2024 had no approved claims.

**Table 1:** Annual chiropractic‑related compensation claims decided by NPE, 2013–2024

Year	Rejected (*n*)	Approved (*n*)	Approved (%)	Total (*n*)
2013	13	1	7	14
2014	12	2	14	14
2015	8	1	11	9
2016	13	2	13	15
2017	3	1	25	4
2018	10	1	9	11
2019	6	4	40	10
2020	15	1	6	16
2021	8	3	27	11
2022	15	1	6	16
2023	12	0	0	12
2024	15	0	0	15
Overall	130	17	12	147

Legend: Annual counts of completed, rejected, and approved compensation claims related to chiropractic consultations 2013–2024

### Claim frequency by chiropractic consultations

The total number of consultations between 2013 and 2024 was 24.3 million, with an average of 2 million consultations per year (median 2.1 M; range 2.2 M ~ 1.9 M). The annual rate of approved claims per 100,000 consultations was 0.1 (median 0.1; range 0-0.2). When restricting exposure to first-assessment consultations (K1) the rate averaged 0.5 per 100 000 (median 0.4; range 0-1.4) (Table 2).

**Table 2:** Annual chiropractic consultations and compensation claims in Norway, 2013–2024

Year	First-assessment consultations (K1)	Follow-up (K2)	All consultations (K_tot)	Claims (*n*)	Approved claims (AC)	Proportion of approved claims (all consultations) AC/100K_Ktot	Proportion of approved claims (first-assessment consultations) AC/100K_K1
2013	239,009	1,659,825	1,898,834	14	1	0.05	0.42
2014	241,817	1,652,258	1,894,075	14	2	0.11	0.83
2015	239,303	1,612,211	1,851,514	9	1	0.05	0.43
2016	246,787	1,635,380	1,882,167	15	2	0.11	0.81
2017	247,182	1,784,303	2,031,485	4	1	0.05	0.40
2018	271,006	1,816,091	2,087,097	11	1	0.05	0.37
2019	278,987	1,877,627	2,156,614	10	4	0.19	1.43
2020	266,340	1,725,518	1,991,858	16	1	0.05	0.38
2021	296,304	1,863,149	2,159,453	11	3	0.14	1.01
2022	307,994	1,808,521	2,116,515	16	1	0.05	0.32
2023	327,024	1,810,908	2,137,932	12	0	0	0
2024	335,112	1,783,865	2,118,977	15	0	0	0
Overall	3,296,865	21,029,656	24,326,521	147	17	0.07	0.52

Legend: Values are presented as counts and rates per 100,000 consultations; additional decimal precision is shown for very small values

### Injury types associated with claims (NPE internal codes)

Categories displaying high approval rates were “Bleeding/thrombosis/hematoma”; 60% (3/5 approved) and “Central nervous system disturbance”; 60% (3/5 approved), and two standalone case categories read as administrative codes (680 and 657). The most frequent categories were “Treatment not performed correctly”; 38% (*n* = 56) and “Pain”; 25% (*n* = 37) (Table 3).

**Table 3:** Distribution and approval rates of chiropractor-related compensation claims by NPE injury code

NPE code	Total (*n*)	Approved (*n*)		Approved (%)
680 Psychological distress	1	1	100	
657 Insufficient documentation	1	1	100	
606 CNS disturbance	5	3	60	
640 Bleeding/Hematoma	5	3	60	
620 Local nerve injury	7	2	29	
630 Tissue damage	11	2	18	
650 Functional disorders	6	1	17	
647 Incorrect treatments	56	3	5	
660 Pain	37	1	3	
646 Inadequate treatment/follow-up	10	0	0	
648 Treatment should not have been performed	4	0	0	
604 Circulatory disturbance	1	0	0	
671 Delayed diagnostics	1	0	0	
690 Other/unknown	1	0	0	
645 Delayed treatments	1	0	0	

Legend: Claims are ranked by approval proportion. Values are shown as counts (n) and percentages

### Presenting diagnoses and injury codes (ICD‑10)

Presenting diagnostic codes were available for all 147 claims and were predominantly musculoskeletal. Musculoskeletal diagnoses (ICD-10, chapter M) accounted for 131/147 cases (89%), with 15 approved claims (15/131, 11%). Most of the approved musculoskeletal claims were associated with back and neck pain presentations. In addition, one approved claim involved thoracic pain, one multifocal pain presentation, and one case coded as cauda equina syndrome.

Presentations from other ICD-10 chapters were infrequent. General symptoms (ICD-10, chapter R), which included headache and pelvic pain presentations, accounted for seven cases (7/147, 5%), with one approved claim (1/7, 14%). Nervous system disorders (ICD-10, chapter G) consisted primarily of headache diagnoses, with no approved claims. Injuries to specific body regions (ICD-10, chapter S) included three cases (3/147, 2%), of which one concussion-related claim was approved (1/3, 33%). Additional isolated presenting diagnoses were observed for jaw disorders (ICD-10, chapter K), malignant neoplasms (ICD-10, chapter C), and eye and ear disorders (ICD-10, chapter H), none of which resulted in approved claims (Table 4).

**Table 4:** Presenting diagnoses and approval rates by truncated ICD-10 codes and ICD-10 chapters

ICD-10 code	Rejected (*n*)	Approved (*n*)	Approved (%)	Total (*n*)
C7: Malignant neoplasm of meninges	1	0	0	1
G4: Episodic and paroxysmal disorders	2	0	0	2
H9: Diseases of the ear and mastoid process	1	0	0	1
K0: Diseases of oral cavity, salivary glands and jaws	2	0	0	2
M1: Joint disorders	1	0	0	1
M2: Acquired deformities of fingers and toes	4	1	20	5
M4: Dorsopathies	4	0	0	4
M5: Other dorsopathies	96	14	13	110
M6: Soft tissue disorders (calcific tendinitis)	1	0	0	1
M7: Soft tissue disorders (extremity pain)	8	0	0	8
M9: Other osteopathies and chondropathies	2	0	0	2
R1: Symptoms and signs involving the digestive system and abdomen	1	1	50	2
R2: Symptoms and signs involving the skin and subcutaneous tissue	2	0	0	2
R5: General symptoms and signs	3	0	0	3
S0: Injuries to the head	0	1	100	1
S1: Injuries to the neck	1	0	0	1
S8: Injuries to the knee and lower leg	1	0	0	1
Overall	130	17	12	147

ICD-10 chapters	Rejected (n)	Approved (n)	Approved (%)	Total (n)
G – Nervous system	2	0	0	2
H – Eye/ear disorder	1	0	0	1
M – Musculoskeletal	116	15	13	131
R – General	6	1	17	7
S – Injury to specific body part	2	1	50	3

Legend: Presenting diagnostic codes were available for all 147 compensation claims. Diagnoses are shown using truncated ICD-10 codes at the subchapter level and further summarised by ICD-10 chapters. *Rejected (n)* and *Approved (n)* indicate the number of rejected and approved claims within each diagnostic category. Approval rates represent the proportion of approved claims within each category

### Injury diagnoses (ICD-10 injury codes)

ICD-10 injury codes were available for 111 of the 147 compensation claims (76%). Injury diagnoses were most frequently classified as musculoskeletal injuries (ICD-10, chapter M; 41/111, 37%), although relatively few of these claims were approved (3/41, 7%). Circulatory system injuries (ICD-10, chapter I) accounted for 16 cases (16/111, 14%) and were associated with the highest number of approved claims (6/16, 38%). Injuries coded as complications (ICD-10, chapter T) represented 32 cases (32/111, 29%), none of which were approved.

Neurological injury diagnoses (ICD-10, chapter G) were infrequent and included one approved claim. Injuries to specific body parts (ICD-10, chapter S), including fractures and nerve injuries, accounted for 11 cases (11/111, 10%), of which three were approved (3/11, 27%). Other injury categories occurred rarely and contributed minimally to the overall distribution of approvals (Table 5).

**Table 5:** Injury diagnoses and approval rates by truncated ICD-10 codes and ICD-10 chapters among claims with available injury coding

ICD-10 code	Rejected (*n*)	Approved (*n*)	Approved (%)	Total (*n*)
G4: Episodic and paroxysmal disorders	1	0	0	1
G8: Paralytic syndromes	1	0	0	1
G9: Disorders of the nervous system	0	1	100	1
H9: Diseases of the ear and mastoid process	3	0	0	3
I6: Cerebrovascular diseases	3	3	50	6
I7: Diseases of arteries, arterioles, and capillaries	7	3	30	10
J9: Other and unspecified disorders of the circulatory system	0	1	100	1
M0: Inflammatory-/septic arthritis	1	0	0	1
M2: Acquired deformities of fingers and toes	9	0	0	9
M4: Dorsopathies	2	0	0	2
M5: Other dorsopathies	22	2	8	24
M7: Soft tissue disorders	3	0	0	3
M8: Other osteopathies and chondropathies	1	1	50	2
R2: Symptoms and signs involving the skin and subcutaneous tissue	1	0	0	1
R4: Symptoms and signs involving cognition, perception, emotional state, and behaviour	1	0	0	1
R5: General symptoms and signs	2	0	0	2
S1: Injuries to the neck	0	1	100	1
S2: Injuries to the thorax	5	0	0	5
S3: Injuries to the abdomen, lower back, lumbar spine, and pelvis	2	2	50	4
S5: Injuries to the elbow and forearm	1	0	0	1
T0: Injury of unspecified part of trunk, limb, or body region	1	0	0	1
T8: Complications of surgical and medical care, not elsewhere classified	1	0	0	1
T9: Sequelae of injuries, of poisoning, and of other consequences of external causes	30	0	0	30
Overall	97	14	13	111

ICD-10 chapters	Rejected (n)	Approved (n)	Approved (%)	Total (n)
G – Nervous system	2	1	33	3
H – Eye/ear disorder	3	0	0	3
I – Circulatory	10	6	38	16
J – Respiratory	0	1	100	1
M – Musculoskeletal	38	3	7	41
R – General	4	0	0	4
S – Injury to specific body part	8	3	27	11
T – Complication	32	0	0	32

Legend: ICD-10 injury codes were available for 111 of 147 compensation claims (76%). Injury diagnoses are presented using truncated ICD-10 codes at the subchapter level and aggregated by ICD-10 chapter. *Rejected (n)* and *Approved (n)* indicate the number of rejected and approved claims within each injury category. Approval rates represent the proportion of approved claims within each category. Percentages based on small numbers of claims should be interpreted with caution

### Basis for NPE approval decisions

The most common reasons for claim rejection, based on internal NPE decision codes, were “no causation” (98/147, 67%) and “no failure in treatment/assessment” (32/147, 22%). All claims recorded as involving a “failure in treatment” (15/147, 10%) were approved. In addition, one claim classified as a “technical failure” and one claim falling under the statutory exception provision were approved. The technical failure concerned a fall from the treatment bench resulting in a clavicular fracture.

Recorded specification of what had failed was explored using case notes and indicated that five cases involved failures to refer the patient to appropriate care, five cases had inadequate documentation, four lacked a specific description of the treatment failure, and three concerned treatment that should not have been provided. Review of free-text case notes showed that all approved claims involving head and neck vascular events referenced the temporal relationship between the chiropractic consultation and symptom onset as part of the causation assessment. The documented interval between consultation and symptom onset ranged from 1 to 10 days.

### Aggregated compensation sum

Total compensation during the study period amounted to 21.7 million NOK (~ 2.2 million EUR), with a mean payout of 1.3 million NOK and a median of 485,000 NOK. The difference between mean and median indicates a right-skewed distribution driven by a small number of high-cost cases.

## Discussion

Out of 24.3 million chiropractic consultations in Norway between 2013 and 2024, only 147 claims were filed, of which 17 (12%) were approved. The most common contributing factors for overall claim approval were failure to refer the patient appropriately and inadequate documentation. Two thirds of the approved claims were neck- and head-related circulatory or central nervous system injuries. These findings are broadly consistent with earlier Scandinavian research, which has also reported very low claim rates (0.5–2.5/100,000 total consultations) and low approval rates (6–16%) in chiropractic care [17, 18]. Norway’s stable rate is contrasted by Denmark’s gradual increase over time [18]. This difference likely reflects variations in compensation criteria between the Norwegian fault-based system and the Danish no-fault system. In no-fault systems, compensation does not depend on demonstrating provider fault (e.g., exceeding a 50% likelihood threshold), which may lower the threshold for claim approval and contribute to higher reported claim rates. The diagnostic profile of Norwegian chiropractic claims (Table 4) reflected their clinical scope, where non-specific spinal complaints is the prevailing category [22, 23]. In Norway, chiropractors function as primary-contact providers with direct-access status, referral rights to specialist healthcare services (e.g., medical specialist consultations and diagnostic imaging), and authority to certify sickness absence for up to 12 weeks within their scope of practice for neuro-musculoskeletal conditions. The low volume of chiropractic compensation claims contrasts with that of general practitioners (GP) (2011–2017: 3,526 cases, 24% approval rate) [24]. Between 2011 and 2017 Norwegian GPs had 87.7 million ordinary consultations, meaning that their claims rate was 4 per 100,000 [16]. For context, 26% of the Norwegian population have annual primary care visits related to musculoskeletal disorders [25]. Norwegian primary care numbers represent general practitioners, physiotherapists, and chiropractors with state reimbursement agreements. However, direct comparisons between general practitioners and chiropractors are limited by variations in patient conditions, diagnostic complexity, consultation volume, and reporting practices. Norwegian claims research for physiotherapy is currently unavailable.

Exploration of free-text comments made by NPE personnel indicated that temporal proximity between the chiropractic consultation and symptom onset was a key consideration in the assessment of approved head and neck vascular claims, with recorded onset occurring between 1 and 10 days after the consultation. Importantly, this reflects the statutory decision-making framework of the NPE rather than implying pathophysiological causation [19]. The possible causal relationship between chiropractic care and such injuries has been extensively explored and does not currently indicate increased risk of such events [13, 26, 27]. Unfortunately, due to small number of cases, no further sub-group analysis of neck- and head vascular injures was possible for this dataset. Most claims were rejected due to “no causation”, and a substantial fraction were deemed within acceptable clinical practice. This emphasizes that many claimants experienced symptom exacerbation or serious events temporally associated with care, but without evidence that the chiropractor’s actions were below the expected standard or that the alleged injury was more likely than not caused by the treatment [19]. This aligns with the clinical complexity of musculoskeletal pain and the natural course of several common and often overlapping conditions such as multisite musculoskeletal pain, disc herniations, degenerative disorders, or headache syndromes, which may progress or fluctuate independently of care, and are considered multi-factorial [28–32].

## Strengths and limitations

Strengths of the current study include comprehensive national registries covering all completed NPE decisions from 2013 to 2024. Another strength is the combination of claims data with national consultation volumes, allowing calculation of claim rates per relevant exposure. The study also benefited from detailed coding which allowed a nuanced analysis of injury categories and approval patterns using both internal NPE coding and ICD-10 codes. The chiropractic activity database is of high quality and is likely to capture most reimbursed chiropractic consultations.

Several limitations must be acknowledged. First, NPE data represent compensation claims, not all adverse events. Many patients who experience adverse events may not pursue compensation claims, and others may file claims without having experienced a treatment-related injury. Thus, these data cannot be used to estimate the incidence or prevalence of adverse events in chiropractic practice. Second, first-assessment consultations plausibly involve greater diagnostic uncertainty than follow-up visits, this is based on clinical reasoning rather than direct empirical validation. Third, ICD-10 diagnostic coding was introduced progressively and was not systematically applied before 2015, limiting temporal consistency for diagnostic subgroup analyses. Fourth, the dataset was assessed by one author (CB) to preserve claimant privacy legislation. Finally, the dataset lacks granular detail on specific treatment techniques, clinical decision-making contexts, or risk factors, preventing procedure-level risk estimation.

## Conclusion

Compensation claims for chiropractic consultations in Norway are rare. Between 2013 and 2024, only 147 compensation claims were filed for 24.3 million consultations and only 17 were approved. Approvals were most common in cases where a temporal relationship between the consultation and the injury could be established. Most claims were rejected due to lack of causation. These findings contribute contemporary Norwegian data to an international discussion on patient safety and provide a baseline for future monitoring and quality‑improvement efforts.

## Data Availability

The datasets analysed during the current study are not publicly available due to claims case confidentiality but are available from the corresponding author on reasonable request, pending approval from the data owner (NPE).

## Abbreviations

EUR: European Euro (currency)
ICD-10: International Classification of Disease – tenth revision
KUHR: Norwegian national chiropractic consultation data
NOK: Norwegian Krone (currency)
NPE: Norwegian System of Patient Injury Compensation
